# Comparison of Urinary Tract Infection Rates Associated with Different Catheterization Methods Following Major Pelvic or Abdominal Surgery: A Bayesian Network Meta-Analysis of Randomized Controlled Trials

**DOI:** 10.3390/life16020280

**Published:** 2026-02-05

**Authors:** Thanh-Nhan Doan, Thi-Hong-Phuc Le, Li-Wei Chou

**Affiliations:** 1 Department of General Surgery, Vinmec Da Nang International Hospital, Vinmec Healthcare System, Hanoi 11622, Vietnam; drchannhan@gmail.com; 2 Faculty of Health Sciences, Mekong University, Vinh Long 85000, Vietnam; 3 Department of Traditional Medicine and Rehabilitation, Nghe An Oncology Hospital, Vinh City 460000, Vietnam; bslephucungbuou@gmail.com; 4 Specialist Level I in Rehabilitation Medicine, Hanoi Medical University, Ha Noi 100000, Vietnam; 5 Department of Physical Medicine and Rehabilitation, China Medical University Hospital, Taichung 404327, Taiwan; 6 Department of Physical Therapy and Graduate Institute of Rehabilitation Science, China Medical University, Taichung 404328, Taiwan; 7 Department of Physical Medicine and Rehabilitation, Asia University Hospital, Asia University, Taichung 413305, Taiwan

**Keywords:** indwelling Foley catheterization, suprapubic catheterization, intermittent catheterization, postoperative, urinary tract infection, randomized controlled trial, bladder rehabilitation

## Abstract

Background: Postoperative bladder drainage is commonly required following major pelvic or abdominal surgery. Existing evidence indicates substantial variation in urinary tract infection (UTI) risk across different catheterization methods. However, the comparative effectiveness of indwelling Foley catheterization (IFC), suprapubic catheterization (SPC), and intermittent catheterization (IC) remains uncertain. Methods: We conducted a Bayesian network meta-analysis of randomized controlled trials (RCTs) to compare UTI incidence associated with different urinary drainage methods, including IFC, SPC, and IC. PubMed and the Cochrane Library were searched to identify eligible RCTs published from January 2010 to November 2025. Trials comparing at least two of the three catheterization routes following major pelvic or abdominal surgery were included. The primary outcome was the rate of UTI. A Bayesian network meta-analysis with a random-effects model was conducted using the gemtc package in R 4.5.1 and RStudio2026.01.0. The quality of evidence was evaluated using the GRADE approach. Results: Ten RCTs involving 1242 patients met the eligibility criteria. Both IC and SPC demonstrated a reduced risk of UTI compared with IFC. Based on indirect evidence, SPC was not associated with a statistically significant reduction in postoperative UTI compared with IC, with considerable uncertainty in the effect estimate (OR = 0.53, 95% CrI 0.09–2.69). Overall, IC and SPC showed favorable trends in reducing catheter-related complications compared with IFC. Conclusions: This network meta-analysis suggests that SPC and IC may be more effective than IFC in reducing the risk of postoperative UTI following major pelvic or abdominal surgery. However, further high-quality randomized controlled trials that integrate urinary drainage methods with bladder rehabilitation interventions are needed to identify the optimal management strategy for this patient population.

## 1. Introduction

Postoperative bladder drainage is commonly required following major pelvic or abdominal surgery, yet all catheterization techniques carry a measurable risk of urinary tract infection (UTI) [1,2]. This complication is clinically important because it prolongs hospitalization, increases healthcare expenditure, delays recovery, and negatively affects patients’ quality of life [3,4]. Existing evidence indicates considerable variation in UTI risk across different catheterization methods. Bladder drainage remains the cornerstone of postoperative urinary management, with several approaches frequently employed: indwelling Foley catheterization (IFC), suprapubic catheterization (SPC), and intermittent catheterization (IC) [5,6].

In daily clinical practice, the IFC remains the most widely used technique, largely because it has long been integrated into routine care, is technically straightforward, and is familiar to most healthcare providers [7,8]. However, accumulating evidence suggests that this method is associated with a higher incidence of UTI, making the selection of an optimal drainage strategy increasingly challenging [9]. Although some clinical guidelines recommend intermittent catheterization as a means of reducing postoperative complications, including UTIs, the current evidence base remains limited and insufficient to support consistent, evidence-based recommendations for postoperative bladder management [10,11,12]. Suprapubic catheterization has also been proposed as a potential alternative due to its lower UTI risk compared with transurethral catheters. However, its greater invasiveness and higher risk of procedure-related complications have restricted broader adoption [13].

Overall, the available literature is fragmented, with small sample sizes and heterogeneous study designs, highlighting the need for a comprehensive Bayesian network meta-analysis to clarify the comparative risk of UTIs associated with IFC, SPC, and IC in the postoperative setting. To address this knowledge gap, we conducted a systematic review and network meta-analysis to evaluate and compare postoperative UTI rates across these three drainage techniques.

## 2. Materials and Methods

### 2.1. Protocol and Registration

This study was conducted in accordance with the guidance of the Preferred Reporting Items for Systematic Reviews and Meta-Analysis (PRISMA) extension statement for network meta-analysis of healthcare interventions [14] and was registered in PROSPERO: CRD420251272248 (Supplementary Materials).

### 2.2. Eligibility Criteria

Eligible studies were selected based on predefined inclusion and exclusion criteria. Randomized controlled trials (RCTs) published in English from January 2010 to November 2025 were included. Participants were required to have undergone major pelvic or abdominal surgery in which temporary urinary catheterization is routinely used to prevent postoperative urinary retention. Patients with a history of pelvic radiation, previous major pelvic surgery, preexisting urethral abnormalities, neurogenic bladder, voiding dysfunction, or chronic dependence on urinary catheterization were excluded. In addition, patients with indwelling percutaneous nephrostomy tubes or ureteral stents for benign ureteral conditions, such as ureteral stones or ureteral strictures, were also excluded. Studies had to report data on the incidence of UTI in patients with a catheterization duration of ≤5 days, and full-text articles had to be available for review.

Studies were excluded if they were conducted in populations not undergoing surgery. In addition, non-randomized study designs were excluded, including conference abstracts, letters, editorials, guidelines, reviews, commentaries, protocols, clinical observations, case reports, cohort studies, case–control studies, and other non-interventional investigations such as cross-sectional or retrospective surveys. Studies with incomplete data or unreported outcomes were also excluded.

### 2.3. Search Strategy and Study Selection

The literature search was conducted across two major databases, PubMed and the Cochrane Library, using a combination of Medical Subject Headings (MeSH) and free-text terms. The following keywords were applied: “urinary tract infections”, “bacteriuria”, “intermittent catheterization”, “clean intermittent”, “Suprapubic Catheterization”, “suprapubic tube”, “Urinary Catheterization”, “indwelling catheter”, “transurethral catheter”, “urethral catheter”, “post-operative”, “surgical”, “Pelvic Surgery”, “Abdominal Surgery”, “gynecologic”, and “gynaecologic”. These terms were combined using Boolean operators (AND/OR) to maximize the retrieval of relevant studies. The detailed search strategy is presented in Appendix A, Table A1.

Searches were limited to the period from January 2010 to November 2025 and to articles published in the English language. Retrieved records were imported into Zotero (version 7.0.27, Zotero Foundation, Fairfax, VA, USA) for management and screening by the study team: duplicates were removed, titles and abstracts were screened, and full texts were reviewed to identify studies that met the predefined inclusion criteria for the network meta-analysis.

### 2.4. Risk of Bias Assessment

The risk of bias for the included randomized controlled trials was independently assessed by two reviewers using the Revised Cochrane Risk of Bias Tool for Randomized Trials (RoB 2.0). The evaluation covered five key domains: (1) bias arising from the randomization process, (2) bias due to deviations from intended interventions, (3) bias due to missing outcome data, (4) bias in measurement of the outcome, and (5) bias in selection of the reported result. Each study was categorized as having a low risk of bias, some concerns, or high risk of bias according to the RoB 2.0 criteria. Any discrepancies between reviewers were resolved through discussion or consultation with a third reviewer.

### 2.5. Statistical Analysis

Network meta-analysis was performed using the gemtc package in R (version 4.5.1; R Foundation for Statistical Computing, Vienna, Austria) within RStudio (version 2026.01.0, Build 392; Posit Software, PBC, Boston, MA, USA) under a Bayesian framework. A network meta-analysis was conducted and assessed using Markov Chain Monte Carlo (MCMC) simulation. A Bayesian framework was adopted for the network meta-analysis. Unlike traditional frequentist approaches, which rely primarily on point estimates and confidence intervals, the Bayesian method provides posterior distributions of treatment effects by combining observed data with prior assumptions. This framework enables a coherent synthesis of direct and indirect evidence within a single model, yielding probabilistic interpretations of treatment effects and rankings.

Given the relatively sparse and unbalanced treatment network in this study, the Bayesian approach was considered appropriate for generating stable and clinically interpretable estimates. Models were run with four Markov chains, an adaptation (burn-in) of 5000 iterations, 20,000 simulation iterations, a thinning interval of 1, and a random-effects assumption. For dichotomous outcomes, treatment effects are presented as odds ratios (ORs) with corresponding 95% credible intervals (95% CrI). The deviation information criterion (DIC) served as the basis for our decision on whether to use a fixed-effect or a random-effect model. We selected a model with lower DIC values. However, if the DIC values of the two models were close and their difference was within 5, a random-effect model could be chosen. Gelman–Rubin diagnostic, along with a review of trace plots, would be used to assess convergence. A network plot was generated to visualize the available comparisons to visualize multiple comparisons. For treatment ranking, the surface under the cumulative ranking curve (SUCRA), which ranges from 0% to 100%, was calculated. When comparing the various therapies, the intervention with the highest SUCRA value had the highest likelihood of being the best one. Network graphs and related figures were generated using the netgraph function and ggplot2 in RStudio.

### 2.6. Procedure for Evaluation and Analysis

The entire systematic review process, including the development of the search strategy, study screening, data extraction, risk of bias assessment, and result analysis, was con-ducted by the author team. Any inconsistencies were resolved through discussion among the authors until a consensus was reached.

## 3. Results

### 3.1. Selection of RCTs

**Figure 1:**
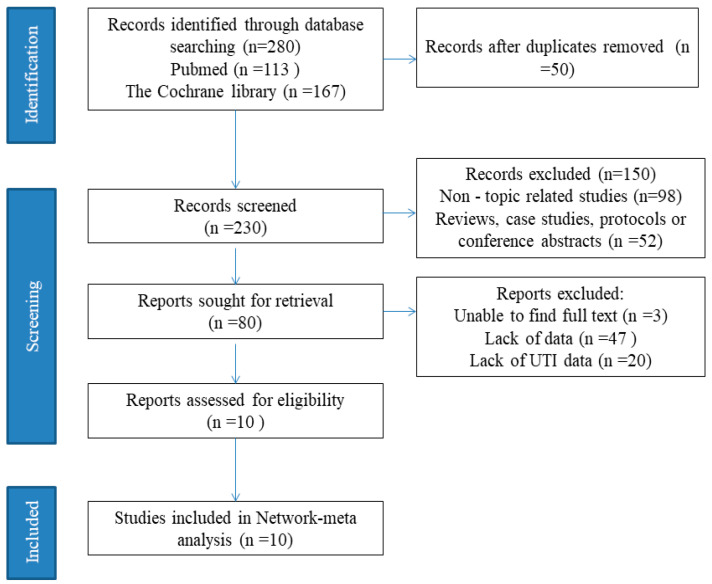
Flow diagram of the study selection process.

Out of the 280 titles that were examined, 230 records had their abstracts evaluated. From this group, 80 complete studies were assessed to determine their eligibility. Eventually, a total of 10 publications, involving 1242 patients from 9 countries, were conducted from January 2010 to November 2025, and met the inclusion criteria for this analysis. We obtained the 280 titles and abstracts from two sources such as PubMed and Cochrane Library. After removing duplicate articles, a quick review helped eliminate irrelevant articles. We then proceeded to evaluate the eligibility of full articles. The final selection consisted of 10 studies for quantitative synthesis [15,16,17,18,19,20,21,22,23,24]. This process is illustrated in the PRISMA flow diagram (Figure 1).

### 3.2. Study Characteristics

An overview of the study characteristics is presented in Table 1, summarizing the randomized controlled trials included in the network meta-analysis and published between January 2010 and November 2025. Most trials enrolled patients undergoing pelvic or urogenital surgeries, particularly pelvic organ prolapse and stress urinary incontinence procedures, while a smaller number involved abdominal or orthopedic surgeries. The included studies were conducted in England, Germany, The Netherlands, Pakistan, Sweden, the United States, China, France, and Belgium, and reported comparable outcomes. The sample size of the RCTs ranged from 48 to 208 participants. The evaluated interventions included intermittent catheterization (IC) in 6 studies (*n* = 225), indwelling foley catheterization (IFC) in 10 studies (*n* = 579), and suprapubic catheterization (SPC) in 4 studies (n = 264).

### 3.3. Quality Assessment

**Figure 2:**
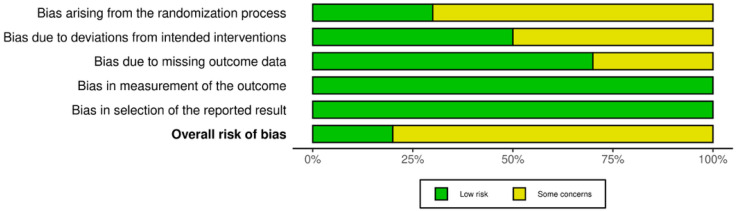
Risk of bias graph, presented as a percentage across all included studies.

**Figure 3:**
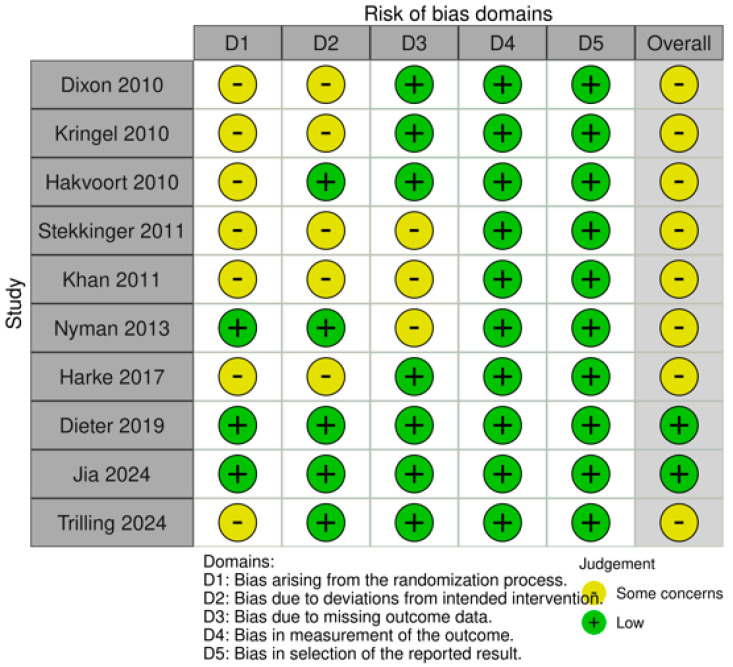
Risk of bias summary for the included studies [15,16,17,18,19,20,21,22,23,24].

All included trials (10/10) were two-arm studies. The risk of bias for the included RCTs is illustrated in Figure 2 and Figure 3. Risk of bias assessment was conducted by the study authors in accordance with the Cochrane RoB 2.0 tool for all 10 RCTs, covering five key domains: deviations from intended interventions (D2), missing outcome data (D3), outcome measurement (D4), and selective reporting (D5). Overall, the studies demonstrated low risk of bias, with minimal potential bias in the randomization process. All studies were judged to have low risk of bias in outcome measurement and selective reporting. Approximately 50% of studies were rated as having a low risk of bias for deviations from intended interventions. No RCT was judged to be at high risk of bias, and three RCTs were considered at some concerns for missing outcome data. Additionally, five RCTs were rated as having some concerns regarding deviations from the intended interventions. The certainty of the evidence was assessed using the GRADE approach, based on five domains for downgrading: risk of bias, inconsistency, indirectness, imprecision, and publication bias. Each outcome was rated as high, moderate, low, or very low certainty. The full assessment is presented in Table 2.

### 3.4. Network Evaluations

**Figure 4:**
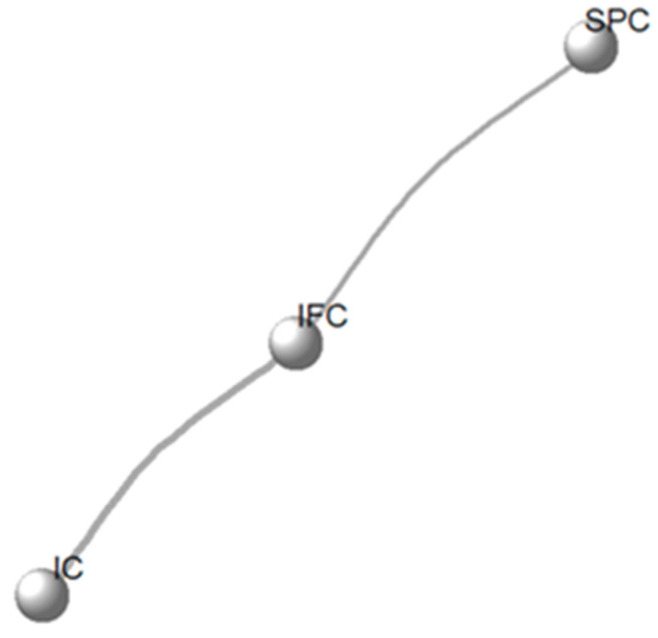
Network plot of included treatments in the network meta-analysis.

The network plot illustrates the evidence structure for the three catheterization methods. All interventions—IC, IFC, and SPC—were connected, indicating that direct or indirect comparisons are available among them. The simple three-node linear network shows that the evidence base is relatively sparse, with only one comparison linking each pair of interventions. This connected network enabled the synthesis of direct and indirect evidence, although the evidence base was sparse for performing a network meta-analysis. However, it also highlights that the overall strength of evidence may be limited by the small number of studies contributing to each link. Figure 4 illustrates the evidence network describing the interventions tested in RCTs for their effectiveness in reducing UTI incidence.

### 3.5. Model Convergence Assessment

Convergence of the Markov Chain Monte Carlo (MCMC) simulations was evaluated using the Gelman–Rubin diagnostic. The potential scale reduction factor (PSRF) was 1.000711, indicating excellent convergence and suggesting that the chains were well-mixed and the results are reliable.

**Figure A1:**
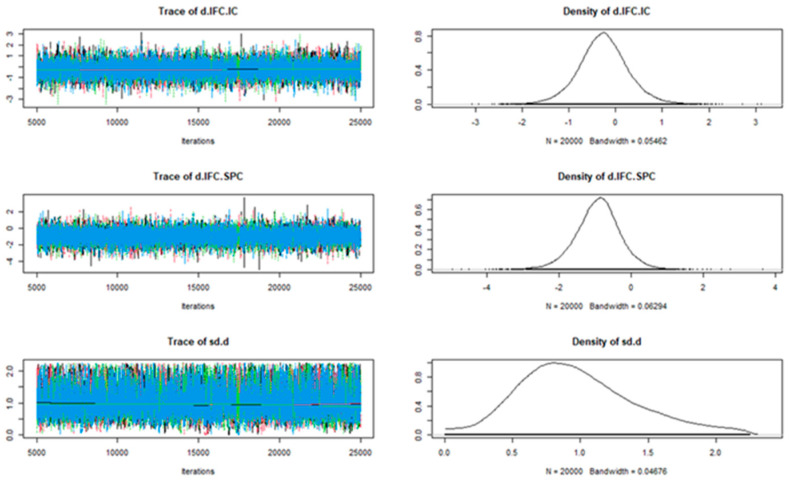
Trace plots for convergence assessment of the Bayesian network meta-analysis model for urinary tract infection (UTI) incidence.

**Figure A2:**
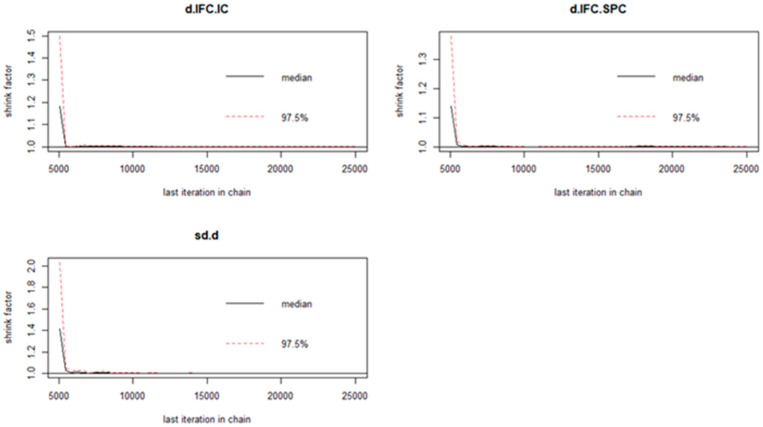
Posterior density plots of treatment effects for urinary tract infection (UTI) incidence.

The trace plots for all monitored parameters (d.IFC.IC, d.IFC.SPC, and sd.d) demonstrated good mixing and stable oscillation around a constant mean without visible trends or drifts. The corresponding posterior density plots showed smooth, unimodal distributions with no evidence of multimodality or irregularities, suggesting well-behaved posterior estimates. For both treatment effect parameters (d.IFC.IC and d.IFC.SPC), the posterior distributions were centered near zero, implying that the comparative effects between groups were small and may not be clinically meaningful. Overall, both the trace and density plots support that the MCMC simulation reached convergence and produced reliable posterior estimates for subsequent inference. The Brooks–Gelman–Rubin shrink factor plots for all parameters (d.IFC.IC, d.IFC.SPC, and sd.d) demonstrated rapid convergence. Across all parameters, the shrink factor started above 1.1 during the early phase of sampling but quickly dropped toward 1.0 as the chains mixed, remaining stably at 1.0 for the remainder of the iterations. Both the median and the 97.5th percentile curves approached 1.0 without further fluctuation, indicating that between-chain variance and within-chain variance had equalized. These results confirm that all monitored parameters achieved satisfactory convergence, supporting the reliability of the posterior estimates and the robustness of the Bayesian model inference. The trace and density plots are presented in Figure A1 and Figure A2 of Appendix A.

### 3.6. Assessment of Network Consistency

Global consistency was evaluated by comparing the Deviance Information Criterion (DIC) between the consistency and inconsistency models, while local consistency was assessed using node-splitting analysis. The DIC values were similar (absolute ΔDIC = 0.005441881), indicating good consistency within the network.

### 3.7. Results of Network Meta-Analysis for Indirect Comparisons Between Interventions

The relative treatment effect matrix shows the pairwise comparisons among IC, IFC and SPC on a log-odds scale. When compared with IFC, IC demonstrated an estimated effect of −0.27 (95% CrI, −1.37 to 0.86), indicating no statistically significant difference but suggesting a possible trend toward lower outcome risk. SPC, when compared with IFC, showed an even larger negative effect estimate of −0.90 (95% CrI, −2.24 to 0.31), implying a potentially greater reduction in risk, although the credible interval also crossed the null. For the comparison between IC and SPC, the effect size was 0.64 (95% CrI, −0.99 to 2.41), indicating that IC may be associated with a higher risk relative to SPC; however, the wide and imprecise credible interval reflects substantial uncertainty. Overall, none of the pairwise comparisons reached statistical significance; however, the direction of effect consistently favors SPC as the potentially most effective strategy, followed by IC, with IFC performing the worst across comparisons. The relative treatment effects are shown in Table 3.

**Figure A3:**
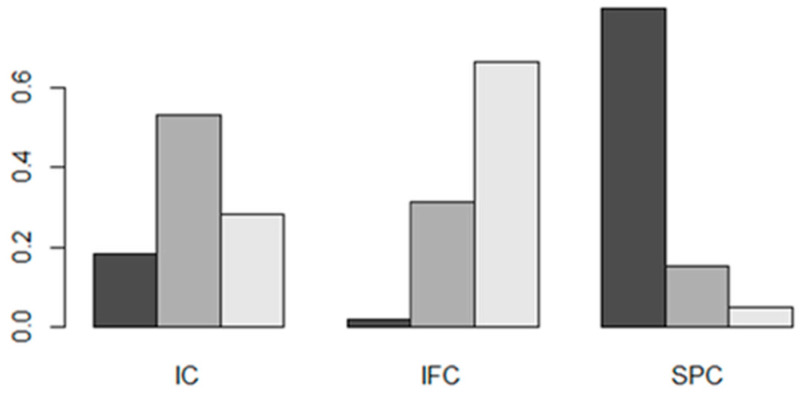
Posterior rank probabilities for each catheterization method.

**Figure A4:**
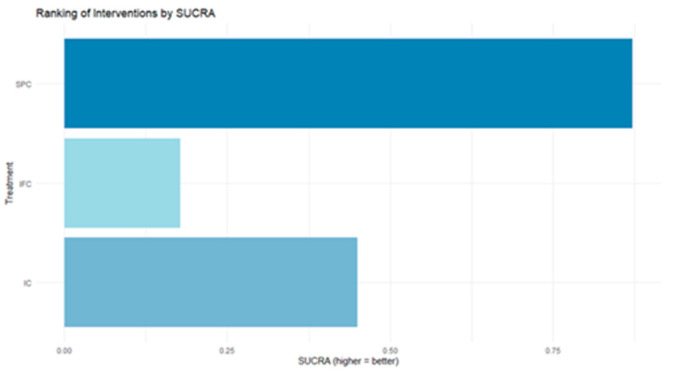
Ranking plot of interventions based on SUCRA values for reducing UTI incidence.

Figure A3 illustrates the posterior rank probabilities for the three catheterization strategies. For IC, the probabilities were relatively balanced across the middle ranks, indicating moderate uncertainty regarding its relative performance. IFC showed a higher probability of occupying the worst rank, suggesting that it is more likely to be the least effective option. In contrast, SPC demonstrated the highest probability of being ranked first, with markedly lower probabilities for the lower ranks, indicating that SPC is the most likely to be the best-performing intervention among the three. Overall, the ranking distribution supports SPC as the preferred strategy, whereas IFC is consistently the least favorable. The posterior rank probabilities are shown in Figure A3. The ranking results are summarized in Table A2, and the rank probability plot is presented in Figure A4 of Appendix A.

## 4. Discussion

To the best of our knowledge, this is the first Bayesian network meta-analysis to comprehensively evaluate strategies for preventing or mitigating postoperative UTIs in patients undergoing major abdominal or pelvic surgery between January 2010 and November 2025. By synthesizing evidence from 10 RCTs, this study provides the most up-to-date and robust comparison of IFC, SPC, and IC in the context of short-term bladder drainage.

Previous systematic reviews and meta-analyses have reported higher rates of UTI among patients managed with IFC compared with SPC or IC, particularly in gynecological and urological surgical populations that require short-term bladder drainage [7,25]. However, an updated Cochrane review in 2015 concluded that the available evidence remained insufficient to draw definitive conclusions regarding the comparative risk of symptomatic UTI among IFC, SPC, and IC [26], highlighting the persistent lack of consensus in this field. Notably, the Cochrane review reported a trend toward a reduced risk of UTI with SPC. Although the included studies shared similarities in design, substantial heterogeneity existed regarding UTI diagnostic criteria as well as the timing and frequency of urine sampling across trials. Postoperative UTI represents a distinct clinical entity, as indications for catheterization and catheter dwell time differ from those in other hospitalized populations. Moreover, the routine use of perioperative antibiotic prophylaxis may alter urine culture results or mask clinical manifestations of UTI. Despite these differences, meta-analytic approaches remain appropriate to address this long-standing controversy, as conducting a single RCT directly comparing all three bladder drainage strategies would be extremely challenging, if not infeasible, due to the large sample sizes required and the inherent heterogeneity of surgical populations. By including RCTs that applied varying UTI definitions but ultimately informed clinical treatment decisions, our analysis aimed to reflect clinically significant UTIs, i.e., infections warranting treatment in real-world practice.

Consequently, there exists substantial variability not only in UTI definitions but also in diagnostic approaches across studies. The included UTIs comprised both symptomatic and asymptomatic cases, increasing the risk of misclassification: some clinically symptomatic infections may yield negative urine cultures, while some asymptomatic patients may have positive cultures. Although this broad inclusion represents a limitation, it simultaneously enhances the generalizability of the findings. It should be emphasized that antibiotic therapy is recommended only for symptomatic bacteriuria. Postoperative patients represent a particularly complex population, as many experience baseline or transient bladder dysfunction related to surgical intervention, and catheter-related irritative symptoms may further complicate accurate diagnosis. In line with current international guidelines, routine antibiotic prophylaxis for the prevention of catheter-associated UTI is not recommended in patients with short-term indwelling urinary catheters. Guidelines from the Infectious Diseases Society of America, the Centers for Disease Control and Prevention, the European Association of Urology, and NICE consistently emphasize that antimicrobial therapy should be reserved for clinically symptomatic UTI rather than asymptomatic bacteriuria. Consistent with these principles, postoperative antibiotic use was not routinely reported in the included randomized controlled trials, reflecting real-world, guideline-based practice [27,28]. To maximize statistical power and facilitate the simultaneous comparison of multiple bladder drainage strategies, we conducted a network meta-analysis that integrated both direct and indirect comparisons through a common comparator within a Bayesian hierarchical framework. All included data were derived from RCTs in which the catheterization duration was ≤5 days.

In contrast to the study by Han et al., our findings demonstrate that IFC was associated with a higher risk of UTI compared with SPC and IC, whereas Han et al. reported no statistically significant difference between SPC and IC in the context of short-term catheterization [5]. In our Bayesian network meta-analysis, both SPC and IC showed trends toward lower UTI risk compared with IFC; however, the certainty of evidence remained low due to wide credible intervals, and differences did not reach statistical significance.

The observed benefits of SPC are biologically plausible. Suprapubic access avoids transurethral manipulation, thereby reducing mucosal injury and limiting the introduction of periurethral microorganisms into the bladder. Avoiding continuous contact between the catheter surface and the dense microbial flora of the periurethral and vaginal regions, which are major sources of ascending infections, may further reduce the risk of infection. SPC may provide particular advantages in patients anticipated to require prolonged bladder drainage immediately after surgery, as it is associated with lower rates of catheter reinsertion, likely due to the feasibility of performing voiding trials without catheter removal [29]. Placement of SPC intraoperatively under anesthesia is a relatively simple and safe procedure. Moreover, SPC preserves urethral function, facilitates more complete bladder emptying, and reduces post-void residual volume, collectively limiting bacterial proliferation. Improved catheter care and reduced contamination during handling may also contribute to lower infection rates [13,30].

The mechanisms underlying the benefits of IC are well-understood: IC reduces the duration of catheter presence in the urinary tract, thereby limiting bacterial adhesion and biofilm formation. Because the catheter is removed immediately after voiding, mucosal irritation is minimized, bladder emptying is more complete, and exposure to periurethral flora is reduced, factors that collectively explain the lower risk of bacteriuria and symptomatic UTI [12]. Additionally, IC can be taught to patients and/or caregivers prior to discharge, potentially reducing healthcare costs associated with increased nursing care. Our findings further support the 2015 European Association of Urology (EAU) guidelines, which reported lower rates of bacteriuria with IC compared with indwelling catheterization [27]. When compared with IFC, both IC and SPC demonstrated trends toward reduced UTI risk; however, the certainty of evidence was low, mainly due to risk of bias in the included trials and imprecision from wide credible intervals crossing the null effect. The indirect comparison between SPC and IC was based solely on evidence via IFC from 10 RCTs. Although the pooled estimate suggested a lower risk of postoperative UTI with SPC versus IC (OR 0.53; 95% CrI 0.09–2.69), the difference was not statistically significant and accompanied by very wide credible intervals, resulting in very low certainty of evidence due to indirectness and serious imprecision. Therefore, these results should be interpreted as hypothesis generating only.

Beyond catheterization strategy alone, postoperative urinary outcomes may also be influenced by bladder rehabilitation interventions. In this context, structured bladder training plays a crucial role in facilitating early Foley catheter removal and restoring preoperative voiding autonomy. Postoperative bladder training programs including timed voiding, gradual bladder capacity expansion, pelvic floor muscle activation, and sensory retraining may promote detrusor recovery and improve coordination between bladder contraction and sphincter relaxation. Early implementation of these strategies may reduce prolonged catheter dependence, lower catheter-associated UTI risk, and enhance overall functional recovery [31]. Integrating structured bladder rehabilitation into postoperative urinary management may therefore optimize outcomes beyond catheter selection alone. In the future, well-designed, adequately powered RCTs are needed to determine the effectiveness, optimal timing, and key components of bladder training protocols following Foley catheterization.

Several important limitations should be acknowledged. First, the included RCTs did not report downstream urinary drainage related outcomes, such as postoperative hydronephrosis, obstructive uropathy, or the need for secondary urinary diversion. Most trials focused primarily on lower urinary tract outcomes, while data on upper urinary tract complications were lacking. Consequently, the impact of downstream urinary drainage interventions could not be evaluated, and these unmeasured factors may have influenced the observed postoperative infection rates. Second, although suprapubic catheterization was implemented as a predefined randomized intervention in the included RCTs rather than as a rescue procedure, confounding by indication cannot be fully excluded. In real-world clinical practice, suprapubic cystostomy is often selectively applied in patients with anticipated difficulty in urethral catheterization or a higher baseline risk of urinary retention, which may limit the generalizability of the observed benefits, particularly in short-term catheterization settings. Third, methodological heterogeneity across the included studies represents an additional limitation. The trials encompassed heterogeneous surgical populations, including different types of abdominal and pelvic procedures, with variations in study design, patient characteristics, and perioperative management. In particular, the timing of urinary catheter removal and the timing and frequency of postoperative urine sampling differed substantially between studies, contributing to clinical and methodological heterogeneity that may have affected outcome ascertainment. Fourth, at the time of the literature search, only 10 randomized controlled trials met the predefined eligibility criteria for inclusion. Moreover, the evidence network contained no closed loops, precluding the application of node-splitting analyses to formally assess inconsistency between direct and indirect evidence. Further restricting the patient population to specific surgical procedures or standardized perioperative protocols would likely yield even fewer eligible trials and may render a network meta-analysis infeasible. Future high-quality, well-designed RCTs with more homogeneous patient populations are therefore warranted to enable more refined and robust network meta-analytic comparisons. Fifth, our literature search was restricted to studies published in English and was conducted using only two electronic databases (PubMed and the Cochrane Library). As a result, relevant studies published in other languages or indexed exclusively in additional databases may have been missed, and grey literature was not included, introducing a potential risk of publication and language bias. Finally, although a Bayesian hierarchical framework was employed, the evidence network’s limited geometry limits the ability to comprehensively evaluate inconsistencies between direct and indirect evidence. Therefore, the comparative estimates should be interpreted with appropriate caution.

## 5. Conclusions

Overall, this network meta-analysis provides a comprehensive overview of the effectiveness of three interventions across 10 randomized controlled trials in reducing the incidence of UTI. SPC and IC appeared to be the most effective strategies for preventing or mitigating UTI; however, these results should be interpreted with caution. In the GRADE assessment, the certainty of evidence for the outcomes was rated as low, primarily due to risk of bias across the included trials and substantial imprecision, as reflected by wide confidence intervals crossing the null. Given the limited certainty of evidence, definitive clinical decision-making cannot be based on the current findings. Instead, clinical decisions should be individualized, with a focus on planning bladder rehabilitation, including the timing of early catheter removal and tailored bladder training programs for each patient.

## Figures and Tables

**Table 1:** Overview of the basic characteristics of the ten RCTs included.

Study (Author, Year, Country)	Intervention Groups	Sample Size	Event/Number of Rates of UTI	Surgical Procedure	Definition of UTI	Conclusions
Dixon, 2010 [15] England	IC/IFC	13/09	36/36	Women undergoing surgery for pelvic organ prolapse and/or stress urinary incontinence	Preoperative urinalysis for all admissions. A midstream urine sample for culture and sensitivity testing after positive urinalysis results for leukocytes and nitrites. Postoperatively, catheter or midstream urine samples were obtained for culture if UTI was suspected, based on pyrexia >37.5 °C after postoperative day 1, urinary frequency or dysuria, offensive urine, and positive urinalysis for leukocytes and nitrites.	The use of IC following urogynecological surgery is associated with a more rapid return to normal micturition and a shorter hospital stay, although the clinical significance of the difference is perhaps limited.
Kringel, 2010 [16] Germany	SPC/IFC	01/31	32/100	Patients with an indicated anterior colporrhaphy plus an optional further procedure	Urine sample on postoperative day 4, asymptomatic bacteriuria defined using Centers for Disease Control and Prevention definition.	In this trial, the optimal bladder catheterization strategy after anterior colporrhaphy was IFC for 24 h.
Hakvoort, 2010 [17] The Netherlands	IC/IFC	05/13	43/40	Vaginal prolapse repair	Presence of >10^5^ CFU/mL in voided culture obtained upon normalization of post-void residual volume and cessation of catheterization.	IC is preferred over IFC.
Stekkinger, 2011 [18] The Netherlands	SPC/IFC	06/06	64/62	Vaginal prolapse repair	Presence of >l0^4^ CFU/mL in culture.	SPC was comparable to IFC in the prevention of postoperative voiding dysfunction after vaginal prolapse surgery, but it was associated with a higher rate of complications.
Khan, 2011 [19] Pakistan	IC/IFC	04/03	22/26	Internal optical urethrotomy	A growth of >l0^5^ bacteria/mL urine.	IC is a simple and effective way of reducing stricture recurrence after internal optical urethrotomy.
Nyman, 2013 [20] Sweden	IC/IFC	05/13	43/40	Hip surgery	Positive urine culture results at discharge >l0^5^ CFU/mL.	Both IFC and IC are feasible options in clinical practice. Although each method has its advantages and disadvantages, avoiding IFC may reduce unnecessary catheterizations.
Harke, 2017 [21] Germany	SPC/IFC	03/08	59/78	Robot-assisted radical prostatectomy	Positive urine culture results at discharge >l0^5^ CFU/mL.	SPC is associated with significantly lower pain levels during the catheterization period compared with IFC, without compromising long-term functional outcomes.
Dieter, 2019 [22] USA	IC/IFC	09/11	30/47	Surgery for pelvic organ prolapse	Culture-proven UTI was defined as a urine culture with greater than l0^5^ CFU/mL.	Among women using IFC or IC after surgery, no differences were found in catheter-related burden or non–urinary-related postoperative contacts, and UTI rates were similar between groups.
Jia, 2024 [23] China	IC/IFC	51/51	0/0	laparoscopic adnexal surgery	Presence of 100 coliform organisms per ml urine with pyuria (≥10 leukocytes per mm^3^) or ≥10^5^ CFU/mL of any pathogenic organism per mL urine on culture.	No significant difference in complications was observed between the IC and IFC.
Trilling, 2024 [24] France and Belgium	SPC/IFC	109/99	08/11	Surgery for mid and/or lower rectal cancers	Positive urine culture results at discharge >l0^5^ CFU/mL.	IFC should be preferred over SPC in male patients.

Abbreviations: SPC: suprapubic catheterization; IFC: indwelling Foley catheterization; IC: intermittent catheterization; UTI: urinary tract infection.

**Table 2:** Summary of findings for bladder catheterization strategies (IFC, IC, and SPC).

Comparison	No of Participants (Studies)	Relative Effect (OR, 95% CrI)	Absolute Effect	Certainty of the Evidence (GRADE)	Reasons for Downgrading
IC vs. IFC	225 (6 RCTs) [15,17,19,20,22,23]	0.76 (95% CrI 0.25 to 2.36)	195 (95% CrI 74 to 429)	Low	Downgraded for risk of bias and imprecision due to some concerns in four RCTs and a wide 95% CrI (0.25–2.36) crossing the null.
SPC vs. IFC	264 (4 RCTs) [16,18,21,24]	0.41 (95% CrI 0.11 to 1.36)	75 (95% CrI 21 to 212)	Low	Downgraded for risk of bias and imprecision owing to some concerns in all four RCTs and a wide 95% CrI (0.11–1.36) crossing the null.
SPC vs. IC	Indirect comparison via IFC (10 RCTs) [15,16,17,18,19,20,21,22,23,24]	0.53 (95% CrI 0.09 to 2.69)	Not estimable	Very Low	Downgraded for indirect evidence and serious imprecision (95% CrI 0.09–2.69).

Abbreviations: SPC: suprapubic catheterization; IFC: indwelling Foley catheterization; IC: intermittent catheterization; RCT: randomized control trial.

**Table 3:** Relative Effectiveness of Interventions in the rate of UTI.

IC	IFC	SPC
IC	0.27 (−0.86, 1.37)	−0.64 (−2.41, 0.99)
−0.27 (−1.37, 0.86)	IFC	−0.9 (−2.24, 0.31)
0.64 (−0.99, 2.41)	0.9 (−0.31, 2.24)	SPC

Abbreviations: SPC: suprapubic catheterization; IFC: indwelling Foley catheter; IC: intermittent catheterization; UTI: urinary tract infection.

## Data Availability

All data used in this study were extracted from previously published studies. No new data were generated. Data sharing is not applicable to this article.

## Supplementary Materials

The following supporting information can be downloaded at: https://www.mdpi.com/article/10.3390/life16020280/s1, Table S1. PRISMA NMA Checklist of Items to Include When Reporting A Systematic Review Involving a Network Meta-analysis.

## Abbreviations

The following abbreviations are used in this manuscript:

UTI	Urinary Tract Infection
IFC	Indwelling Foley Catheter
SPC	Suprapubic Catheterization
IC	Intermittent Catheterization

## Appendix A

**Table A1:** The complete search strategy and results.

PubMed	((“urinary tract infections”[MeSH] OR “urinary tract infection”[Title/Abstract] OR UTI[Title/Abstract] OR bacteriuria[Title/Abstract]) AND (“Catheterization, Intermittent”[MeSH] OR “intermittent catheterization”[Title/Abstract] OR “clean intermittent”[Title/Abstract] OR CIC[Title/Abstract] OR ISC[Title/Abstract] OR “Suprapubic Catheterization”[MeSH] OR “suprapubic catheter”[Title/Abstract] OR “suprapubic tube”[Title/Abstract] OR SPC[Title/Abstract] OR “Urinary Catheterization”[MeSH] OR “indwelling catheter”[Title/Abstract] OR “transurethral catheter”[Title/Abstract] OR “urethral catheter”[Title/Abstract]) AND (postoperative[Title/Abstract] OR post-operative[Title/Abstract] OR surgery[Title/Abstract] OR surgical[Title/Abstract] OR gynecologic[Title/Abstract] OR “Pelvic Surgery”[MeSH] OR “Abdominal Surgery”[MeSH] OR gynaecologic[Title/Abstract]) AND (randomized[Title/Abstract] OR randomised[Title/Abstract] OR “randomized controlled trial”[Publication Type]))
Cochrane Library	(UTI OR “urinary tract infection”) AND (suprapubic OR “intermittent catheterization” OR “indwelling catheter”) AND (postoperative OR surgery)

**Table A2:** Ranking of interventions based on network meta-analysis estimates for the reduction in rate of UTI.

No.	Treatment	SUCRA	Rank
1	SPC	0.8721313	1
2	IC	0.4495125	2
3	IFC	0.1783562	3
